# Relationship Between T-helper 1 Inflammatory Biomarkers and Hematological Index Responses in Patients With Multiple Sclerosis

**DOI:** 10.7759/cureus.75278

**Published:** 2024-12-07

**Authors:** Zhian Dezayee

**Affiliations:** 1 Basic Sciences, Hawler Medical University, Erbil, IRQ

**Keywords:** biomarkers, cytokines, hematological indices, lipoproteins, multiple sclerosis

## Abstract

Background

Multiple sclerosis is a chronic, progressive, disabling disease associated with a high rate of infection, evidence of chronic inflammation, and a high mortality rate. Abnormalities of serum cytokines and changes in the activity of inflammatory cells were associated with relapsing-remitting multiple sclerosis (MS-RR). This study aims to introduce new inflammatory ratios derived from hematological and lipid indices as discriminators of T-helper (Th)-1/Th-2 activity in RR-MS.

Methods

This cross-sectional study recruited 40 RR-MS patients and 30 healthy individuals. Th-1 and Th-2 cytokines, including interferon-y (INF-γ), tumor necrosis factor-a (TNF-α), interleukin (IL)-4, IL5, and IL-10 measurements, were performed using enzyme-linked immunosorbent assay technique. Hematological indices and lipid profiles were measured as routine laboratory investigations. SPSS Statistics version 20 (IBM Corp. Released 2011. IBM SPSS Statistics for Windows, Version 20.0. Armonk, NY: IBM Corp.) was used for data analysis. An independent sample t-test was used to compare two means, and Spearman correlation was used to assess the correlation between different markers. A p-value of <0.05 was considered statistically significant.

Results

Serum levels of Th-1 and Th-2 cytokines in RR-MS were significantly higher than in healthy subjects (p<0.001). The ratios of INF-γ-to-IL4 and TNF-α-to-IL-10 were significantly lower than the corresponding ratios of healthy subjects (p<0.001). Monocyte-to-high-density lipoprotein ratio (MHDLR) is significantly lower (p<0.001) than healthy subjects (9.4±1.62 vs. 6.4±1.1, respectively) and significantly correlated positively with INF-γ (r=0.417) and TNF-α (r=0.565), while MHDLR inversely correlated with IL-4 (r=-0.445) and IL-5 (r =-0.386). Lymphocyte-to-non-high-density lipoprotein ratio (LNHDLR) is significantly (p<0.001) higher than healthy subjects (18.2±3.3 vs. 13.1±2.2, respectively) and significantly correlated inversely with INF-γ (r=-0.484) and TNF-α (r=-0.456), while LNHDLR positively correlated with IL-4 (r=0.565) and IL-5 (r=0.532). The area under the curve (AUC) of MHDLR mimics the AUCs of Th-1 cytokines, while the AUC of LNHDLR mimics the AUCs of Th-2 cytokines.

Conclusions

MHDLR and LNHDLR served as pro-inflammatory and anti-inflammatory markers, respectively, with profiles similar to TH-1 and Th-2 cytokines. These findings suggest that these lipid-related ratios are non-invasive, cost-effective biomarkers for monitoring RR-MS patients' immune response and inflammatory status.

## Introduction

Multiple sclerosis (MS) is a chronic, progressive, disabling disease associated with a high rate of infection, evidence of chronic inflammation, and a high mortality rate. Cytokine profile in patients with relapsing-remitting multiple sclerosis (RR-MS) characterized by non-significantly high levels of IL-4, IL-10, and TNF-α [1]. Researchers believed that pro-inflammatory cytokines, including tumor necrosis factor-a (TNF-α), interferon-y (INF-γ), interleukin (IL)-1, 12, IL-17, and IL-22, may induce MS [2]. The serum levels of T-helper (Th)-1 lymphocyte cytokines, including TNF-α and INF-γ, are markers of relapse, as high INF-γ and low TNF-α serum levels were observed [3]. Th-2 lymphocytes protect the central nervous system against the neuroinflammation and degeneration of the axon [4]. Cytokines related to the Th-2 activation are IL-4, IL-5, and IL-10. In an experimental autoimmune encephalomyelitis animal model, serum IL-4 was low and a useful prognostic marker [5]. Low serum levels of IL-5 and non-significant changes in the Il-4 and IL-10 suggested that the protective effect of these cytokines is theoretical [6]. Recent studies found a high neutrophil-to-lymphocyte ratio (NLR) in MS patients compared with healthy subjects, with a mean value of 2.12 versus 1.72, respectively [7]. During the relapsing phase, the NLR is higher than in the remitting phase but does not correlate with the Expanded Disability Status Scale (EDSS) [8].

Further studies demonstrated that NLR is further increased when MS patients are exposed to stress [9]. Lymphocyte-to-monocyte ratio (LMR) and platelet lymphocyte ratio were considered inflammatory markers and tended to be altered in different pathological conditions [10,11]. In addition to the cytokine and inflammatory markers involved in MS, alteration of lipid metabolism is also shared in the pathogenesis of MS. Research has shown significant positive correlations between cholesterol and low-density lipoprotein cholesterol with a score of disability in RR-MS [12]. Therefore, an interaction between inflammatory markers derived from hematological indices and lipids with the cytokine levels Th-1/Th-2 as markers of disease activity may occur in relapsing-remitting MS. Limited research has examined such a potential interaction. Therefore, this study aimed to introduce new inflammatory ratios derived from hematological and lipid indices as discriminators of Th-1/Th-2 activity in RR-MS.

## Materials and methods

Setting

This observational cross-sectional study was conducted in Erbil, Kurdistan Region of Iraq, from February to June 2021.

Ethics approval

This observational cross-sectional study included patients with RR-MS who attended the Rizgary Teaching Hospital in Erbil, Kurdistan Region of Iraq, and consultant private clinics for follow-up. The Institutional Scientific Committee of Hawler Medical University approved this study (approval number: 4/9). This study was carried out according to the Helsinki Declaration and the guidance of the ethical committee that stated, "Any medicine, device, or intervention should not be harmful to the patient, and the patient at any time is free to withdraw from the study or to refuse admission into the study." Each patient signed a consent form at the time of entry into the study. The Rizgary Teaching Hospital is the main teaching hospital attached to Hawler Medical University. Administrative approval was obtained from the Rizgary Teaching Hospital for this study.

Participants

This study included patients with RR-MS who attended the Rizgary Teaching Hospital in Erbil, Kurdistan Region of Iraq, and consultant private clinics for follow-up. Eligible patients were both genders, aged less than 45 years old. The criteria for inclusion are patients with a long-standing proven RR-MS. Consultant neurologists diagnosed the patients based on their clinical background, laboratory investigations, and radiological pictures, including nuclear magnetic resonance. The exclusion criteria included pregnancy, lactation, or evidence of serious complications. The authors and the neurologists assessed the disease activity score by applying EDSS. All the patients were treated with different modalities of medicines, including β-interferon, azathioprine, monoclonal antibodies (natalizumab, alemtuzumab, corticosteroids, and lipid-lowering agents), in addition to the symptomatic relieving drugs. Patients with renal failure, chronic liver disease, terminal illness, recent infections, and severe disability were excluded. A total number of 40 patients with RR-MS (15 men and 25 women) with a mean ± SD of age 37.1 ± 4.8 years, and 30 healthy subjects (21 men and nine women) aged 34.3 ± 5.0 years) were included.

Laboratory investigations

Venous blood samples were drawn and divided into two portions. The first portion was added to the anti-coagulant-containing test tubes to determine the hematological indices using the automatic Coulter machine (Beckman Coulter, Inc., Brea, CA, USA). The second portion was added into plain test tubes, and sera were separated by centrifugation for determination of lipid indices, cytokines (TNF-α, INF-γ, IL-4, IL-5, and IL-10), and high-sensitivity C-reactive protein (hs-CRP). Hematological indices included white cell count and differential count, red cell width distribution, mean platelet volume, platelet distribution width, and erythrocyte sedimentation rate (ESR). Lipid indices included cholesterol and high-density lipoprotein cholesterol (HDL-c). The following ratios were calculated: NLR, LMR, platelet-to-lymphocyte ratio (PLR), monocyte-to-high-density lipoprotein (MHDLR), and lymphocyte-to-non-high-density lipoprotein ratio (LNHDLR). Non-HDL-c was determined by subtracting serum HDL-c concentration from serum cholesterol concentration. The Th-1/Th-2 ratio as a marker of disease activity is calculated by dividing each of the Th-1 cytokines (TNF-α and IFN-γ) by each of the Th-2 cytokines (IL4, IL-5, and IL-10).

Statistical analysis

The results were expressed as a number, percentage, and mean ± SD. ESR and hs-CRP as discriminating and dependent variables were statistically assessed using receiving operating characteristics for measuring the area under the curve (AUC) and 95% confidence interval of Th-1 and Th-2 cytokines and the ratios derived from hematological and lipid indices. All calculations and diagrams were made using SPSS Statistics version 20 (IBM Corp. Released 2011. IBM SPSS Statistics for Windows, Version 20.0. Armonk, NY: IBM Corp.). Means and standard deviation were used to present the findings. An independent sample t-test was used to compare the two means. Spearman correlation was used to assess the correlation between different markers. A p-value of <0.05 was considered statistically significant.

## Results

This study included 40 patients (15 men and 25 women) and 30 controls (12 men and 18 women). The mean ± SD of the duration of illness and the score of EDSS for the 40 patients were 6.1 ± 1.6 years and 3.28 ± 0.69, respectively. Table 1 shows the measurements of Th-1 and Th-2 cytokines. Serum levels of Th-1 cytokines were significantly higher than the corresponding levels of healthy subjects. The serum level of TNF-α accounted for 5.77 folds of the serum level of INF-γ in RR-MS patients. Th-2 cytokines, including IL-4, IL-5, and IL-10 of RR-MS patients, were significantly higher than corresponding levels of healthy subjects (Table 1). RR-MS patients had a higher serum level of IL-10 than those of IL-4 and IL-5. The ratio of INF-γ to each Th-2 interleukin was significantly higher among RR-MS II patients compared with the corresponding ratios of healthy subjects. A significantly high ratio of TNF-α-to-IL-10 (p<0.001) and a significantly low ratio of TNF-α-to-IL-4 (p<0.001) were observed in RR-MS patients compared with healthy individuals.

**Table 1:** Serum levels of Th-1 and Th-2 markers The results are expressed as mean ± SD. The p-value was calculated using an independent two-sample t-test (two-tailed). INF-γ: interferon-y, TNF-α: tumor necrosis factor-a, IL: interleukin, SD: standard deviation

Determinants	Group I (control) (n=30)	Group II (patients) (n=40)	p-value
Th-1 markers			
INF-γ (pg/ml)	1.24 ± 1.03	18.6 ± 4.4	<0.001
TNF-α (pg/ml)	22.91 ± 5.37	107.4 ±1 8.8	<0.001
Th-2-markers			
IL-4 (pg/ml)	8.55 ± 2.77	19.4 ± 4.0	<0.001
IL-5 (pg/ml)	6.65 ± 2.07	33.7 ± 9.9	<0.001
IL-10 (pg/ml)	5.93 ± 2.19	43.3 ± 10.3	<0.001
Th-1/Th-2 ratio			
INF-γ/IL-4	0.165 ± 0.162	1.04 ± 0.42	<0.001
INF-γ/IL-5	0.207 ± 0.186	0.62 ± 0.28	<0.001
INF-γ/IL-10	0.236 ± 0.200	0.43 ± 0.13	<0.001
TNF-α/IL-4	3.0 ± 1.00	5.94 ± 2.16	<0.001
TNF-α/IL-5	3.80 ± 1.53	3.54 ± 1.41	0.486
TNF-α/IL-10	4.41 ± 1.85	2.47 ± 0.69	<0.001

Table 2 shows that the inflammatory markers (including ESR and hs-CRP) and those derived from hematological indices were significantly higher in RR-MS patients than in healthy subjects.

**Table 2:** Inflammatory markers related to the hematological and lipid indices The results are expressed as mean ± SD. The p-value was calculated by an independent two-sample t-test (two-tailed). ESR: erythrocyte sedimentation rate, hs-CRP: high-sensitivity C-reactive protein, NLR: neutrophil-to-lymphocyte ratio, LMR: lymphocyte-to-monocyte ratio, PLR: platelet-to-lymphocyte ratio, MHDLR: monocyte-to-high-density lipoprotein ratio, LNHDLR: lymphocyte-to-non-high-density lipoprotein ratio, SD: standard deviation

Determinants	Group I (control)	Group II (patients)	p-value
	(n=30)	(n=40)	
ESR (mm/h)	11.0 ± 5.6	31.1 ± 6.5	<0.001
hs-CRP (mg/L)	0.7 ± 0.4	3.8 ± 1.0	<0.001
NLR	1.90 ± 0.14	2.18 ± 0.27	<0.001
LMR	10.1 ± 0.88	66.29 ± 8.40	<0.001
PLR	63.4 ± 13.0	79.76 ± 10.80	<0.001
MHDLR	9.4 ± 1.62	6.4 ± 1.1	<0.001
LNHDLR	18.2 ± 3.3	13.1 ± 2.2	<0.001

Table 3 shows significant inverse correlations between TNF-α with each of IL-4 (r=-0.608, p<0.001) and IL5 (r=-0.541, p<0.001) and between INF-γ with each of IL-4 (r=-0.725, p<0.001) and IL5 (r=-0.729, p<0.001). NLR, MHDLR, and CRP significantly correlated with TNF-α (r=0.446, p=0.004; r=0.567, p<0.001; and r=0.564, p<0.001, respectively) and INF-γ (r=0.470, p<0.001; r=0.417, p=0.008; and r=0.567, p<0.001, respectively) in a positive direction and with IL-4 (r=-0.550, p<0.001; r=-0.445, p=0.004; and r=-0.619, p<0.001, respectively) and IL-5 (r=-0.485, p=0.002; r=-0.386, p=0.014; and r=-0.621, p<0.001, respectively) in an inverse direction. In contrast, LMR inversely and significantly correlates with IL-5 (r=-0.325, p=0.041). The ESR is the only inflammatory marker positively correlated with Th-1 cytokines and, inversely, with the Th-2 cytokine panel used in this study.

**Table 3:** Correlations between Th-1 and Th-2 markers with inflammatory markers related to the hematological and lipid indices The results are expressed as Spearman correlation factor (above value) and p-value (lower value). ESR: erythrocyte sedimentation rate, hs-CRP: high-sensitivity C-reactive protein, NLR: neutrophil-to-lymphocyte ratio, LMR: lymphocyte-to-monocyte ratio, PLR: platelet-to-lymphocyte ratio, MHDLR: monocyte-to-high-density lipoprotein ratio, LNHDLR: lymphocyte-to-non-high-density lipoprotein ratio, INF-γ: interferon-y, TNF-α: tumor necrosis factor-a, IL: interleukin

Markers	TNF-α	INF-γ	IL-4	IL-5	IL-10	NLR	LMR	PLR	MHDLR	LNHDLR	hs-CRP
INF-γ	0.608										
	<0.001										
IL-4	-0.608	-0.725									
	<0.001	<0.001									
IL-5	-0.541	-0.729	0.663								
	<0.001	<0.001	<0.001								
IL-10	0.179	0.133	0.077	0.086							
	0.27	0.414	0.637	0.596							
NLR	0.446	0.47	-0.55	-0.485	-0.008						
	0.004	<0.001	<0.001	0.002	0.962						
LMR	-0.042	0.011	-0.094	-0.325	-0.283	-0.138					
	0.797	0.946	0.562	0.041	0.076	0.395					
PLR	0.103	0.244	0.23	-0.158	-0.168	0.484	0.147				
	0.527	0.128	0.153	0.33	0.3	0.002	0.366				
MHDLR	0.567	0.417	-0.445	-0.386	0.134	0.376	-0.394	0.122			
	<0.001	0.008	0.004	0.014	0.408	0.017	0.012	0.452			
LNHDLR	-0.456	-0.484	0.565	0.532	0.113	-0.597	-0.116	-0.418	-0.346		
	0.003	0.002	<0.001	<0.001	0.487	<0.001	0.476	0.007	0.021		
hs-CRP	0.564	0.567	-0.619	-0.621	0.162	0.533	0.12	0.322	0.695	-0.497	
	<0.001	<0.001	<0.001	<0.001	0.317	<0.001	0.46	0.043	<0.001	0.001	
ESR	0.522	0.545	-0.548	-0.603	-0.041	0.468	0.315	0.479	0.567	-0.548	0.858
	0.001	<0.001	<0.001	<0.001	0.8	0.002	0.047	0.002	<0.001	<0.001	<0.001

**Figure 1:**
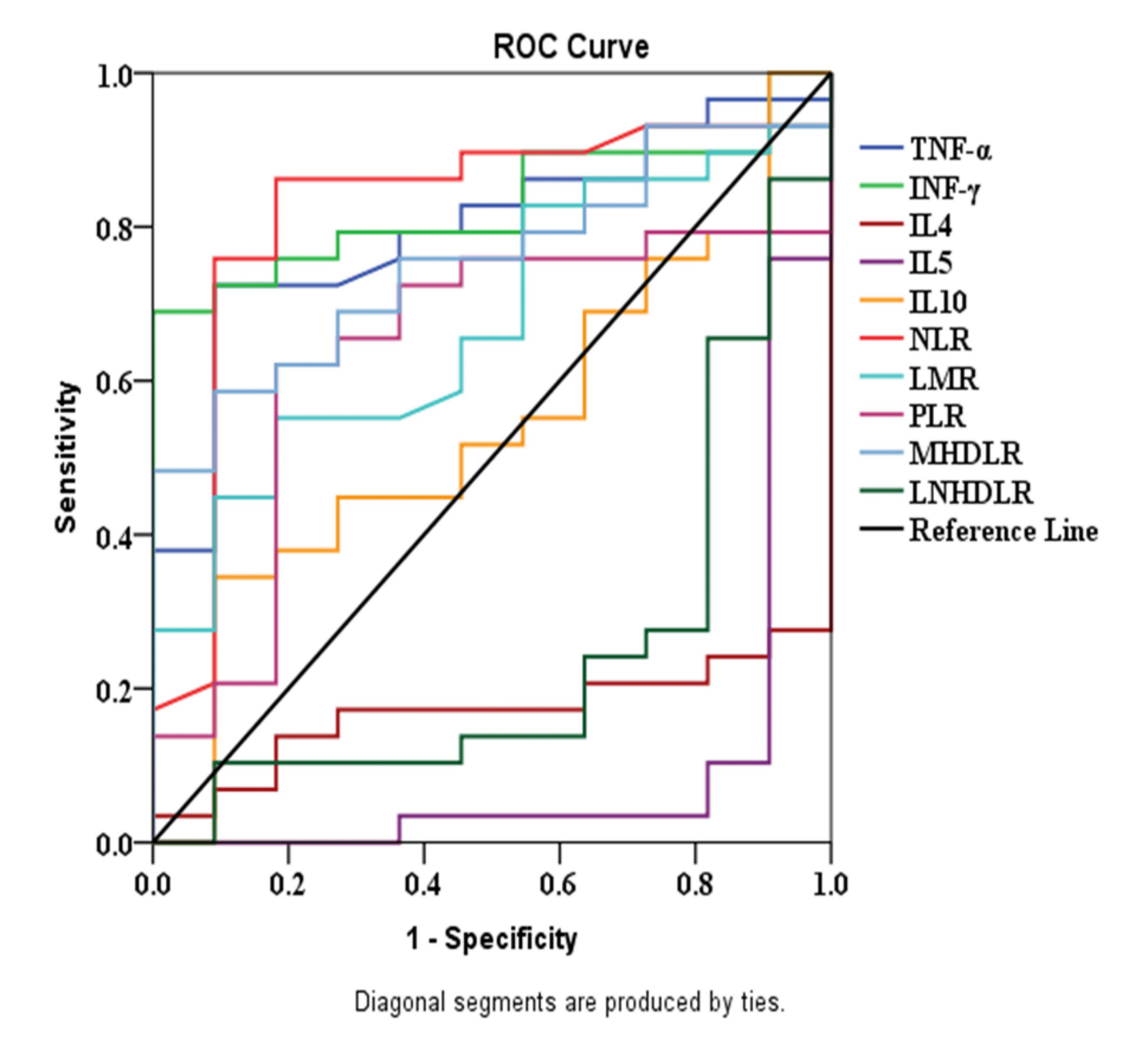
AUC of the Th-1 and Th-2 cytokines, inflammatory indices, and ratios derived from hematological and lipid indices taking the cutoff level of hs-CRP > 3 mg/L as a discriminator ROC: receiver-operating characteristic, NLR: neutrophil-to-lymphocyte ratio, LMR: lymphocyte-to-monocyte ratio, PLR: platelet-to-lymphocyte ratio, MHDLR: monocyte-to-high-density lipoprotein ratio, LNHDLR: lymphocyte-to-non-high-density lipoprotein ratio, INF-γ: interferon-y, TNF-α: tumor necrosis factor-a, IL: interleukin, hs-CRP: high-sensitivity C-reactive protein, AUC: area under the curve

Figure 1 shows the AUC of the TNF-α, INF-γ, MHDLR, and NLR was significantly higher, while the AUC of IL-4, IL-5, and LNHDLR was significantly low, taking the serum level of hs-CRP >3 mg/dl as a discriminator. On the other hand, the AUC of the INF-γ, NLR, and MLR was significantly higher, while the AUC of IL-4, IL-5, and LNHDLR was significantly low, taking the level of ESR >25 mm/hour as a discriminator.

## Discussion

This study showed significantly higher serum Th-1 and Th-2 cytokine levels in RR-MS patients than in healthy subjects. The ratios of TNF-α to each Th-2 cytokine significantly differed from the ratios of INF-γ to each Th-2 cytokine compared with corresponding ratios of healthy subjects. Ratios derived from hematological and lipid indices served as pro-inflammatory and anti-inflammatory markers similar to the cytokine profile. The results of this study agreed with the earlier studies that Th-1/Th2 cytokines were altered in RR-MS [13]. Ratios of INF-γ to the cytokines of Th-2 are higher than those of healthy subjects, indicating that the anti-inflammatory cytokines of Th-2 failed to antagonize the INF-γ and active or relapsing course of RR-MS [14]. The ratio of INF-γ-to-IL-10 is lower than that of INF-γ-to-IL-4 or IL-5, indicating that the production of IL-10 in RR-MS is higher than IL-4 or IL-5. There is evidence that there is a correlation between the production of IL-10 and the severity of a patient's disability, and this explains the low EDSS score found in this study [15].

Moreover, lower ratios of INF-γ and TNF-α to IL-10 indicate that IL-10 can antagonize the cytokines of Th-1, and the patients were in the remitting phase. These changes are unrelated to other confounding factors, including the therapeutic regimen [16,17]. Therefore, the serum IL-10 level serves as a marker that indicates disease remission. Higher ESR, hs-CRP, and NLR values found in RR-MS confirmed earlier studies that explained the role of inflammatory processes in RR-MS. Hematological indices, including LMR and PLR, that served as inflammatory markers significantly increased and supported earlier studies that showed that these markers are useful in assessing inflammatory disease [18,19].

Moreover, we found that the significant changes of MHDLR and LNHDLR took a reciprocal pattern compared with corresponding ratios of healthy subjects, indicating that these ratios are specific for RR-MS. A literature review of the earlier studies did not show any report about the link between these ratios and MS. The ratio of MHDLR served as an inflammatory marker in the subclinical inflammatory conditions [20]. Table 3 discloses the significant positive correlation of MHDLR with Th-1 markers and a significant negative correlation with IL-4 and IL-5, indicating that MHDLR is a pro-inflammatory marker. At the same time, the LNHDLR served as an anti-inflammatory marker, as it positively correlated with IL-4 and IL-5 and negatively correlated with Th-1 cytokines. This observation highlights that a high number of lymphocytes or high levels of HDL favor remission, while a low lymphocyte number and high level of non-HDL favor relapse.

Moreover, this observation explains why statins and natalizumab are used to manage MS [21,22]. Figure 1 confirmed that the AUC of the MHDLR and NHDLR is similar to the AUC of Th-1 and Th-2 cytokines, respectively. The study's strength is using ratios derived from the lipid indices with the inflammatory cells to show the state of relapse or remission.

Study limitations

This study has some limitations. With a cross-sectional design used in the current study, we cannot ascertain causality in associations. The sample size was small, and the participants were selected from one setting. These factors restrict the generalizability of the study findings to the entire study population.

## Conclusions

An imbalance between Th-1 and Th-2 cytokines characterizes RR-MS, reflecting its immunopathology. MHDLR and LNHDLR were identified as surrogate markers for systemic inflammation and immune modulation, with MHDLR showing pro-inflammatory tendencies (Th-1-like) and LNHDLR reflecting anti-inflammatory tendencies (Th-2-like). These lipid-related ratios offer non-invasive, cost-effective biomarkers for assessing immune response, monitoring disease activity, and potentially predicting progression in RR-MS. Larger, longitudinal studies are needed to confirm their clinical utility and explore their role in the immunometabolic landscape of MS.
